# Next-Generation Sequencing Applications for the Study of Fungal Pathogens

**DOI:** 10.3390/microorganisms10101882

**Published:** 2022-09-21

**Authors:** Shiman Jiang, Yanfei Chen, Shengyi Han, Longxian Lv, Lanjuan Li

**Affiliations:** 1 State Key Laboratory for Diagnosis and Treatment of Infectious Diseases, National Clinical Research Centre for Infectious Diseases, Collaborative Innovation Centre for Diagnosis and Treatment of Infectious Diseases, The First Affiliated Hospital, Zhejiang University School of Medicine, 79 Qingchun Rd., Hangzhou 310003, China; 2 Jinan Microecological Biomedicine Shandong Laboratory, Jinan 250021, China

**Keywords:** NGS, fungi, detection, drug resistance, technical innovation

## Abstract

Next-generation sequencing (NGS) has become a widely used technology in biological research. NGS applications for clinical pathogen detection have become vital technologies. It is increasingly common to perform fast, accurate, and specific detection of clinical specimens using NGS. Pathogenic fungi with high virulence and drug resistance cause life-threatening clinical infections. NGS has had a significant biotechnological impact on detecting bacteria and viruses but is not equally applicable to fungi. There is a particularly urgent clinical need to use NGS to help identify fungi causing infections and prevent negative impacts. This review summarizes current research on NGS applications for fungi and offers a visual method of fungal detection. With the development of NGS and solutions for overcoming sequencing limitations, we suggest clinicians test specimens as soon as possible when encountering infections of unknown cause, suspected infections in vital organs, or rapidly progressive disease.

## 1. Introduction

Next-generation sequencing (NGS), which is also named high-throughput sequencing (HTS) [1], provides new ways of detecting microorganisms beyond microbial culture-based methods. NGS was groundbreaking and introduced a reversible stop-codon determination and achieved sequencing by synthesis as a PCR- and GeneChip-based DNA sequencing technique. In the last decade, increasing attention has been paid to NGS’s multiple strengths in exploring nucleic acids [2]. In addition to its accuracy and rapidity, this high-throughput technology shows improved sensitivity and massive information compared to the first generation of DNA sequencing.

Although the current NGS technique is widely used in scientific research, the application in clinical pathogen detection remains to be further strengthened [3]. Earlier identification methods, such as pathogen isolation, selective culture, and pathological examination, are time-consuming and imprecise. After the two days to two weeks required for the culture of pathogenic microorganisms, clinical specimens may show no definite results [4]. However, NGS’s most recently developed microbial identification technology is much less time-consuming. From receiving clinical samples to completing data analysis, the reported turnaround time of NGS is 6 h to 7 days, depending on the sequencing technologies, bioinformatics analyses, and other methods applied [5]. Currently, the main obstacles to the application of NGS for clinics are high diagnostic costs and a lack of expertise in genomics.

After the attention to human-associated bacteria over a long period, human-associated fungi have gradually attracted considerable attention. The generalizability and rapidity of fungal infections call people’s attention to fungal populations, mainly *Candida*, *Malassezia*, *Penicillium*, etc. Nonetheless, the methods used for studying the human fungal populations can impact the analysis and influence the results [6]. The clinical application of NGS has been spreading rapidly in recent years for several reasons, and whole-genome sequencing (WGS) is becoming the most extensively applied form of NGS. NGS adoption for microbiological detection is becoming mainstream, especially for bacteria, viruses, and other prokaryotes. However, there have been few studies on the application of NGS to detect fungi in the last decade. In other words, the rarity of the application of NGS technologies for clinical fungal detection is an issue that remains to be addressed. As of 2014, the genome sequencing of most bacteria and viruses had been performed by NGS, but large numbers of fungal genomes were still missing. As of 2016, only a few hundred rough fungal genome sequences were available [7]. Due to the limited detection tools for clinical fungal infections and the tremendous deleteriousness of fungal infection, the necessity of the application of NGS in fungal diagnosis should be discussed. Also, the next-generation detection tools and fungal genome database should be further improved.

In this review, we summarize the application of NGS in fungal detection as a new gold standard in clinical microbiology. The applications of NGS for fungal screening, drug resistance detection, and identification in different organs, and the technical innovations regarding using NGS for fungal detection, are described.

## 2. NGS and Fungal Sequencing

It has been almost two decades since NGS was first invented, which marks the beginning of fungal high throughput sequencing. Continuously developing over the past decade, NGS not only acts as a gene sequencing tool but also acts as a microbial function detector. The main sequencing systems of NGS include the 454 sequencing system, the Illumina sequencing system, and the SOLiD system [8]. The NGS method that is now widely used was improved from the existing methods by Daryl M. Gohl’s group. A series of improvements, such as increased template concentrations, a reduced number of PCR cycles, and highly persistent polymerase and proofreading polymerase enzymes, have contributed to the increased accuracy of microbiome studies [9]. The error rate of NGS in sequence splicing is in the range of 0.1–15% [10]. For reducing the sequencing error rate of NGS, a new computational method named SequencErr was invented, which helped improve sequencing accuracy [11]. From a diagnostic perspective, identifying and removing bacterial, fungal, and viral genomic contamination are critical for NGS sequencing. This strategy was validated using DecontaMiner, which can be easily combined with standard NGS procedures to unmap contaminated sequences [12]. K-mer analysis not only allows clinical microbiological detection based on WGS and antimicrobial resistance (AMR) but also may be used for the genetic prediction of antibiotic sensitivity [13,14]. In addition, in terms of data processing, because the NGS platform is able to generate a large amount of sequence data, how to process these data poses a challenge to the bioinformatics analysis [8]. A user-friendly framework known as Orione provides a comprehensive computational pipeline that improves the user experience [15]. We have also compiled some bioinformatics pipelines for NGS data analyses from recent years and listed them in Table 1. 

As a diagnostic technology, NGS should ideally be fast, convenient, accurate, broad-spectrum, and user-friendly, although the cost remains significant. There is still a long way to go to establish a novel, mature microbial detection platform for clinical and scientific testing. NGS has also been increasingly used to help diagnose fungal infections [19]. NGS provides the possibility of exploring fungal populations at the strain level. For example, by exploring *Saccharomyces cerevisiae* strains, researchers found that 13 variable genes could represent almost all of their phylogenetic information. And the same method used in yeasts can also be generalized to other individuals [20]. Studies have confirmed that operational taxonomic units (OTUs) over 500 bp in length are more helpful in studying fungal biomes. Based on the K-mer analysis workflow, a suitable k-mer size facilitates gene assembly for fungal strains [21]. Meanwhile, pool sequencing (iPool-Seq) is a large-scale insertion mutation screening method that improves toxicity factor screening in fungi [22]. We also collated the pros and cons of some NGS-based strategies for fungal pathogen detection in Table 2.

NGS is reported to be applied to some fungal infection-related diseases. First, invasive fungal diseases (IFDs) are local or systematic pathogen invasions responsible for high morbidity and mortality caused by various groups of fungal species, especially *Candida* spp. and *Aspergillus* spp. [28]. Kidd et al. [29] provided a detailed summary of the use of fungal molecular polymerase chain reaction (PCR) assays for investigating many kinds of IFDs, showing that PCR can be used to detect fungal species with reasonable specificity. Then, they suggested that NGS can be used for the discriminative analysis of fungal genetic diversity, including drug resistance identification and outbreak investigation. Second, mucormycoses are deadly IFDs, having high mortality, unavoidable disfiguring surgical treatment, and limited therapeutic options. Vincent M. Bruno et al. made conclusions on Mucormycoses and presented their objective insights. Unbiased NGS technology contributes to the related research of Mucorales, consisting of fungus–host interactions, diagnostic improvement, genome architecture, and others [30]. Moreover, *Trichophyton rubrum* is an opportunistic pathogen responsible for progressively expanding invasive diseases. NGS technology targeting microRNAs showed that the inactivated germinated *T. rubrum* microconidia co-cultured with human macrophages promoted the release of proinflammatory cytokines and changes in the regulation of microRNAs [31]. In addition, the group led by Ana Lúcia Fachin co-cultured *T. rubrum* CBS 118892 with human keratinocytes to assess the efficacy of terbinafine, a medicine used to treat dermatophyte infections [32]. Besides these examples, the first study to determine and evaluate nonmodel fungal genomes by modified NGS was conducted for paracoccidioidomycosis. After systematically improving the original Sanger sequence assembly, several genes critical to virulence and pathogenesis were studied more carefully.

The use of the NGS technology in fungal pathogen detection offers the following benefits: First, NGS technology is suitable for hostile culture and slow-growing microbial infections, such as fungi [33]. It will be a useful tool for low fungal loads [19]. Second, NGS provides more accurate identification of fungal species and is even more specific than other methods [4]. However, on account of technical reasons and objective errors, current NGS technologies are not entirely plausible to some extent. Regarding fungi, Leho Tedersoo [6] and his group provided their opinions and recommendations that many large and small issues are always hidden in the current NGS technology. In addition, both the repeatability of fungal sequencing data and the availability of public data are needed for mycobiome sequencing. Likewise, a research team conducted a study comparing the effect of the Respiratory Pathogen ID/AMR (RPIP) kit on a targeted NGS workflow. After comprehensive contrasts were implemented, they concluded that NGS workflows cannot replace the traditional culture and other techniques. The reason they cannot be replaced is the complexity of the bioinformatic analysis [34]. Moreover, different types of Internal Transcribed Spacer (ITS) primers may tendentiously lead to different types of fungi. For instance, ITS1-F, ITS1, ITS5, et al., have a bias toward basidiomycetes amplification, while ITS2, ITS3, ITS4, et al., are more eccentric to ascomycetes [35]. 

Currently, the applications of NGS, ranging from innovative diagnostic methods to routine clinical detection, are growing in leaps and bounds. Both the cost and the speed of detection have improved, allowing NGS to be used for routine microbial detection [36]. Especially since 2005, both the innovation and evolution of NGS technologies and the reduced costs of the required testing materials have promoted genomic testing [36]. To distinguish a broader spectrum of species, the repeatability, quantification results, and classification accuracy of NGS should be improved. NGS is still far from being implemented in the routine of diagnostic laboratories for fungal infections. Not only the prohibitive expense but also its popularity among clinicians lead to this result. With the advantages of reducing turnaround time, localized pathogen WGS should be sufficiently promoted [37]. The identification of fungi by NGS technology not only has its necessity but also still has room for future development.

## 3. NGS and Antifungal Resistance

NGS makes fungi identification more accessible and more accurate. Besides its application to identify fungal types, NGS also can study anti-fungal resistance and drug targets by building a data library and combining other means. Take *C. albicans* as an example. *Candida* spp. is the most frequent cause of fungal disease in humans. By establishing genetic and epigenetic interspecies networks of different strains of *Candida albicans* and their hosts through two-sided NGS data identification, Yeh et al. [38] extracted core host–pathogen cross-talk networks. In addition to screening and identifying specific fungal types, NGS can be used to assess AMR.

Furthermore, the functional channels and pathogenic and defense mechanisms of *C. albicans* were studied, as were potential drug targets and multiple-molecule drugs for treating *C. albicans* infections [38]. By combining NGS with Fourier transform infrared (FTIR) spectroscopy, 256 phenotypes and genotypes of pathogenic *Candida* strains were identified. Among these strains, *C. albicans* was the easiest to identify by NGS of the nuclear large ribosomal subunit (LSU) and ITS [39]. Colabella et al. [40] screened 286 strains of pathogenic yeasts by NGS targeting the regions of the D1/D2 domains of the LSU and ITS1. Multidrug-resistant *Candida glabrata* was also analyzed for fungal resistance through the Sensititre YeastOne^®^ YO10 assay (TREK Diagnostics, Cleveland, OH, USA). When exposed to the anti-fungal drug fluconazole, *C. albicans* shows a loss of chromosomes and may even become haploid. Chang et al. [41] used NGS and flow cytometry to analyze the DNA contents of fungal strains after fluconazole treatment and even developed a technical NGS platform for analyzing chromosome copy numbers. 

Moreover, NGS can be used in anti-fungal susceptibility testing (AFST) and incorporated into the original method to develop a new approach using nascent technologies. AFST is a viable clinical detection means for determining the minimal inhibitory concentration (MIC). Due to various reasons, such as the emergence of drug-resistant strains or even multiple drug resistance, limited anti-fungal agents, a high turnaround time (TAT), and so on, AFST requires constant optimization, innovation, and promotion. The limitations of the traditional AFST can be solved by NGS or WGS assembly-based approaches. For example, the use of NGS is important in the detection of Candida resistance to azole drugs because mutations are distributed over multiple gene sequences [42].

What’s more, the application of massively sequencing technologies may have the ability to reveal undescribed mutations [42]. During the last few years, NGS has found some inherent variants error in anti-fungal protein-coding genes, lending support to the definition of the anti-infectious effect of innate and adaptive immune collaboration [43]. Regarding fungal resistance detection, NGS has played an essential role in improving the efficacy of anti-fungal agents. For example, micafungin has been certified as a potent anti-fungal agent [44]. The differences in anti-fungal resistance to drugs such as azole, 5-fluorocytosine, and echinocandin conferred by different single-nucleotide polymorphisms (SNPs) have also been assessed by NGS [45].

Moreover, the detection of wild-type gene mutations has been performed via NGS. New types of echinocandin and azole resistance were found by screening 391 SNPs [46]. NGS performed genetic analysis and mutation searches of isolated strains to understand the changes in gene mutation mechanisms during the development of drug resistance in *Aspergillus fumigatus*. In an aspergilloma isolate, researchers identified the deletion of a region covering a total of 11 genes, suggesting that mutations also occur during chronic infection with *A. fumigatus* [47]. To probe the genes of drug resistance and the possible mechanisms, NGS could be adopted to explore the resistant fungi and choose appropriate anti-fungal agents. The development of fungal NGS technology perhaps provides a relevant contribution to the development of anti-fungal drugs.

In addition to using NGS to identify fungal resistance and changes in fungal genetic material after treatment with anti-fungal agents, the analysis of antibiotic-producing fungi can also be performed by NGS. *Streptomyces leeuwenhoekii*, for example, produces novel antibiotics known as polyketones. The combined use of Illumina MiSeq and Pacific Biosciences SMRT (PacBio) technologies to assemble actinomycete replicators into single contigs has been recommended by researchers as an experimental pipeline [48]. The “mutagenesis to uncover targets by deep sequencing” approach has identified mutations related to benomyl and rapamycin resistance [49]. By sequencing the ITS rRNA region for the identification of fungi, scientists may have opportunities to find the fungi which owned the excreted or contained active ingredient.

All in all, the application of NGS contributes a lot to the research of anti-fungal resistance, such as anti-fungal resistance and drug targets, anti-fungal drugs, antibiotic-producing fungi, and so on. Hence, with the constant development of NGS technologies, efforts still need to be made continuously.

## 4. NGS and Fungal Infection in Different Organs

According to the site of infection, pathogen infections can be roughly divided into the following categories: respiratory tract infections, central nervous system (CNS) infections, bloodstream infections (BSIs), and local infections. Fungi can cause a range of fungal diseases in different parts of the body, such as the brain, mouth, eyes, ear canal, respiratory tract, digestive tract, vagina, skull, and blood. Because of the low detection rates and long culture times of fungi, routine tests provide no advantages in finding causative organisms hiding in various body parts. Since the first case was reported in 2014, more than 300 cases of fungus infection have been diagnosed by NGS technology [19]. Here, we discuss the application of NGS in the detection of fungi in different body parts and analyze its characteristics in clinical application, including advantages and existing problems.

### 4.1. CNS

All in all, intracranial infections are the most dangerous and urgent compared to other body parts. Despite the high incidence rate, the detection ratios of pathogens are less than half. NGS-based microbiome analysis shows excellent potential for the detection of infectious diseases of the nervous system, which is reflected in the sensitivity of the results, detection speed, and costs [50]. An intracranial fungal infection only accounts for a small percentage of the total intracranial infections, approximately 0.8% [51]. As far as we know, the favorable ratio of fungal culture is not high. 

Additionally, more and more scientific research on intracranial fungal detection is being explored. Alonso et al. performed systematic studies of nervous system diseases, such as Alzheimer’s disease (AD). They recently applied NGS technology to identify specific fungal species in patients with AD. Alonso’s group examined brain samples from nine patients [52]. NGS revealed the existence of some more common fungal genera in AD patients, such as Streptomyces, Greymold, etc. [53]. By identifying fungal species in the brains of HD patients by NGS, Alonso et al. were also the first to show that some fungi, particularly *Ramularia* species, exist in regions within or near the nucleus of the frontal cortex and the striatum. Furthermore, HD patients show a significantly different distribution of fungi in their brains than those with Parkinson’s disease, which suggests that NGS can be used to help find fungal distinctions between different CNS diseases [54]. Notably, a systematic analysis of the application of NGS for encephalitis diagnosis was performed by Julianne R. Brown’s group. This comprehensive assessment concluded that NGS should be seen as a second-line tool, not a first-line tool, in cases of chronic and recurrent encephalitis [55]. Nonetheless, in some cases, NGS should be seen as a first-line tool. For example, for those HIV infection patients with low CD4 counts, all kinds of smear and culture results may be pessimistic despite many attempts. Here is a clinical case that gave hints and warnings to clinicians. Unexpectedly, the compound infection of *cryptococcus* and *streptococcus suis* was finally discovered by NGS technology after repeated conventional attempts [56]. Therefore, immunocompromised patients should take early NGS detection into account.

### 4.2. Eyes and Oral

NGS analysis identified certain fungal genera on the ocular surface of healthy volunteers. Through the ITS2 fungal database, core ocular fungal microbiomes were found and analyzed. Most eyes of healthy volunteers own 11 fungi genera. Different kinds of fungi are located on the ocular surface, among which the most common are *Aspergillus*, *Haematonectria*, *Malassezia*, and *Setosphaeria*. The fungal detection rate by NGS was found to be 73.5%, which was much higher than that by culture-based methods (12.5%) [57]. By collecting samples from the ocular surface or various other sites, researchers have found that certain fungi causing different diseases are present at several sites. In an investigation of endophthalmitis, NGS identified 15 more microbes than were identified by a culture-based method [58]. In a comparison between individuals with fungal keratitis and healthy volunteers, the amplification of the ITS2 region demonstrated that fungal dysbiosis in fungal keratitis varied in terms of both diversity and abundance [59]. 

In recent years, the fungi in the oral environment have increasingly been noticed. Prior studies have shown that fungal infections are common in the oral mucosa, whereas oral *Candida albicans* infection was the most common one. However, the function of them needs further exploration in other oral sites. Lakshman Perera Samaranayake et al. discovered that the weighted mean prevalence (WMP) of primary and secondary fungal infections differed a lot by a meta-analysis. The WMP was 6.3% and 7.5% in the culture-based studies and were increased to 12.5% and 16.0% in the NGS-based studies, respectively [60]. Through 18S rRNA-based ITS2 region analysis, a total of 67 oral fungal species were identified in 27 patients with pseudomembranous oral candidiasis (POC), nearly ten species fewer than those found under normal conditions. The count of *C. albicans* is significantly increased in POC patients, which also shows specific changes in the fungal composition, significantly facilitating the clinical use of anti-fungal drugs. After anti-fungal therapy, the oral fungal species of POC patients gradually recover to a status consistent with that of healthy patients [61]. NGS was applied to distinguish the fungi difference between oral squamous cell carcinoma (OSCC) patients and non-OSCC volunteers. Eventually, the *Malassezia* genus was found to be the special biomarker for the putative diagnosis of OSCC [62]. NGS technology was applied to detect fungal species in the oral cavity, providing valuable microecological information about oral diseases or the relationship between fungi and diseases.

### 4.3. Lungs

In some cases, NGS started a new era in the field of respiratory infections and has become a feasible alternative to traditional culture. NGS can more readily identify fungus components and dynamic changes in the airway and detect cross-kingdom microbial interactions [63]. Respiratory microorganisms are identified by collecting sputum or bronchoalveolar lavage fluid (BALF) samples. Metagenomic NGS has been applied to BALF, particularly for distinguishing *A. fumigatus* from *Nocardia* [64]. After NGS analysis, the species of bacteria, fungi, and viruses and their proportions are further identified. Here, we focus on existing studies using NGS to detect respiratory mycoses. 

Researchers have found that fungi also exist in the respiratory tract of healthy people, but the distribution of fungi varies greatly among people due to environmental exposure and other factors. The distribution of fungi in the ambient air affects the microflora of the respiratory tract, especially *Aspergillus* [65]. NGS can potentially be successfully applied for the analysis of the pulmonary fungal microbiome. ITS1 amplicon sequencing showed that in 91.4% of culture-positive *Blastomyces* specimens, the fungal group was dominated by *Bacillus* [66]. There are a few available studies of fungal colonization in the lungs and related diseases based on NGS technology. One such study was conducted on a 61-year-old patient with severe pneumonia in whom empirical treatment was ineffective before NGS sequencing. The outcome of BALF sequencing revealed three pathomycetes, and subsequent targeted treatment significantly improved the patient’s condition [67]. Cystic fibrosis (CF) is a multifactorial disease in which an individual’s genotype and microbiome are critical factors in disease progression. Françoise and Héry-Arnaud indicated that fungi might be an influential factor in the development of diseases through interactions with other pathogens or genes. By applying culture- and NGS-based methods, they revealed that *Aspergillus fumigatus* was an opportunistic pathogen in CF patients. *Candida* or *Malassezia* spp. were also shown to be involved in the course of the disease [68]. Through fungal rRNA sequencing targeting the ITS2 region and the Phy-Lasso method, *Malassezia* and *Aspergillus* spp. were related to pulmonary exacerbation in CF, and *Scedosporium* spp. was associated with reduced lung function [69]. When it comes to early symptomatic pulmonary infection after lung transplantation, NGS was superior to traditional pathogen detection (88.24% vs. 76.47%) [70]. In patients who underwent hematopoietic cell transplantation with pneumonia, cell-free plasma DNA sequencing (cfDNA-seq) detected infections involving more than a single pathogenic mold (especially non-*Aspergillus* species) in 38 of the 75 patients [71]. In conclusion, NGS has important clinical value in the accurate detection of pulmonary fungal infections.

### 4.4. Blood

When bacteria or fungi invade the bloodstream, they can cause sepsis, bacteremia, or septicemia accompanied by metastatic pyogenic abscesses. NGS can be applied for the early identification of BSIs [72] when traditional culture does not work. Fungal infections may occur in 30% of patients with severe sepsis or septic shock, but only 2–3% can be confirmed via culture-based methods [73]. In studies of such infections, a sepsis-indicating quantifier score can eliminate interference, and cfDNA can be used for unbiased sequence analysis. NGS-based diagnostic methods appear to have clear clinical significance for identifying fungal pathogens in patients with mycosis and patients with negative blood cultures [74]. 

Here we have provided some cases of fungal blood infection for which NGS technology was adopted. In a study of 40 pediatric patients with IFD, fungal pathogens were detected in 7 cases, which showed that cfDNA-seq could play a critical role in diagnosing IFDs, especially BSIs [75]. Mwaigwisya et al. listed the molecular diagnostic tests for sepsis pathogens available on the market and proposed that NGS can be applied to rapidly and accurately diagnose BSIs [76]. *Funneliformis mosseae* was detected in coronary artery plaques from two deceased patients by an NGS-guided technique, and evidence of eukaryotes was found in 14 samples from a total of 15 patients [77]. After isolating fungal nucleic acids from the blood, etc., 24.8% of liver transplantation patients harbor relatively severe fungal infections. Therefore, it is vital to use fast, complete, and accurate NGS-based methods to detect such infections requiring particular care in patients after liver transplantation [78]. The antibiotic treatments affect the pathogens in bloodstream infection, making it tough to achieve the clinical examination. By detecting the blood samples of patients with sepsis in intensive care units, the usage of mNGS considerably improved the number of fungi (*p* = 0.012) compared to conventional culture [79]. Moreover, using ITS sequencing for allogeneic hematopoietic cell transplantation (alloHCT), researchers found that the load of *C. albicans* was significantly high in some blood samples [80].

However, there are some problems with the use of NGS in the fungal infections of blood. First, a big challenge is the presence of large amounts of nucleic acids of human pathogens in the blood. By comparison, pathogen nucleic acid appears too trace [76]. Moreover, fungal cfDNA is rapidly cleared from the bloodstream [75]. In addition, the proportion of fungal reads in the samples differs greatly [80]. Therefore, how to treat and deal with these problems is worth exploring next.

### 4.5. Genitourinary System

Under normal conditions, fungi also exist in the urinary and reproductive tracts. However, the colonization or disruption of the balance of particular fungi can lead to disease. By using this technology, the fungal microbiome is easier to study when interference with the normal colonizing microbiota can be ruled out.

As for the urinary tract, the collection of glandular secretions and urine are more convenient compared to other parts of the human body. Fungi play a particularly important role in lower urinary tract symptoms. Ackerman et al. [81] further optimized the NGS-based testing process for the urinary tract microbiome by improving the DNA extraction method, providing appropriate standard samples, and improving storage and sampling conditions. Their innovative approach was a major breakthrough in the clinical application of NGS. As for the reproductive tract, a large vaginal microbiome database named VIRGO has been established to investigate women’s vaginal health [82]. The NGS results from 89 vaginal microbiome samples from Korean women showed good coherence (range, 86.2–89.7%) with DNA probe assay results. The frequent presence of fungi in the female reproductive tract is reflected in NGS data [83]. 

### 4.6. Others

In addition to the parts of the body mentioned above, other varied body parts can also benefit from NGS sequencing. Even when the site of the infection is examined as a formalin-fixed and paraffin-embedded surgical sample, genetic testing is possible. In a study in which a pathologist’s histopathological assessments of surgical specimens were 100% consistent, fungi were detected by culture-based methods in only 27.3% of specimens. However, in the same paraffin-embedded tissue samples, the correspondence of histopathological morphology with NGS results was 93.2% [84]. Moreover, as a diagnostic tool, NGS has been successfully applied to paraffin-embedded granulomatous lobular mastitis tissue samples, resulting in precise pathogen identification [85]. There have been few studies on upper respiratory tract (URT) fungi in patients with otitis media. Certain sequencing methods, such as ITS region sequencing, are needed to study the associated microflora [86]. In addition, the drainage tube fluid and puncture samples could be detection targets when an infection is suspected.

## 5. Conclusions

In the last decade, the diagnosis of fungal infections has significantly advanced with the advent of novel NGS technology. The application of NGS in the routine clinical detection of pathogens has begun to take shape. However, fungal disease-related pathogen detection is lagging in this context. Culture and identification of fungi can often be difficult and challenging due to harsh cultural conditions and hard culture. Hence, researchers should continue to focus on the application of NGS in the detection of fungi by improving the accuracy, reducing the cost, improving the sampling and detection procedures, and accelerating the clinical application of NGS. A series of NGS systems should be established, including procedures for sample collection, nucleic acid extraction and identification, quality control, and the interpretation of standardized results. Considering the high cost of anti-fungal drugs, the cost of NGS for detecting fungal pathogens is not unreasonable. We suggest that clinicians should use NGS technology more actively when they suspect a fungal infection. Also, the clinical consensus should be established, following in the footsteps of technology. When clinical symptoms indicate the possibility of fungal infection or unknown pathogen identification after culture, NGS technology should be considered and incorporated into clinical diagnosis. Therefore, next-generation sequencing technologies should be implemented early-stage when a patient has a combination of being immunocompromised or immune deficiency or even is first suspected of having an intracranial infection. All in all, we hope that both technical staff and physicians will invest their attention and energy in the development and application of fungal next-generation sequencing, which avoids serious consequences and the high cost of treatment caused by fungal infections. 

## Figures and Tables

**Table 1:** Website-available bioinformatics pipelines for NGS data analyses in recent years.

Name	Foundation	Year	Strength	License	Website	Literature Report
Pal_finder	Galaxy-based	2016	Optimized and simplified microsatellite panel with a user-friendly graphical user interface	Open-source	https://palfinder.ls.manchester.ac.uk	[16]
I-ATAC	/	2017	Provided non-computational scientists with intuitive ATAC-seq data processing methods	Only in academic research	https://github.com/UcarLab/I-ATAC	[17]
Octopus-toolkit	GEO based	2018	Facilitated the analysis of available epigenomic and transcriptomic NGS big data with faster speed and friendly operation interface	GNU General Public License	https://github.com/kangk1204/Octopus-toolkit2	[18]
DecontaMiner	/	2019	Accessed the presence of contaminating data through unmapped sequences	GNU General Public License	https://github.com/topics/decontaminer-pipeline	[12]

**Table 2:** The pros and cons of some NGS-based strategies in fungal pathogen detection.

NGS Technology	Pros	Cons	References
WGS	Allow for determination of fungal species, support fungal taxonomy and dispersal patterns	Time-consuming, requires bioinformatics skills and specialized software and equipment	[23,24]
Transcriptome	It helps to elucidate pathogenicity, host defense mechanisms, phenotypic resistance, and their interactions under various conditions	It is of little use in clinical fungal pathogen detection	[25]
ITS	Is the official barcode for fungi with the highest probability of correct identifications, helpful in fungal community study	(i) Diverse intragenomic ITS sequences in some fungal lineages; (ii) Lack of enough sequence polymorphism	[26,27]

## Data Availability

Not applicable.
